# pulseTD: RNA life cycle dynamics analysis based on pulse model of 4sU-seq time course sequencing data

**DOI:** 10.7717/peerj.9371

**Published:** 2020-07-08

**Authors:** Xin Wang, Siyu He, Jian Li, Jun Wang, Chengyi Wang, Mingwei Wang, Danni He, Xingfeng Lv, Qiuyan Zhong, Hongjiu Wang, Zhenzhen Wang

**Affiliations:** 1 College of Bioinformatics Science and Technology, Harbin Medical University, Heilongjiang, China; 2 College of Computer Science and Technology, Heilongjiang University, Harbin, China; 3 Dalian University of Technology, Dalian, China; 4 Key Laboratory of Tropical Translational Medicine of Ministry of Education, Hainan Medical University, Haikou, China; 5 School of Biomedical Information and Engineering, Hainan Medical University, Haikou, China; 6 College of Science, Heilongjiang University of Science and Technology, Harbin, China

**Keywords:** 4sU-seq, RNA-seq, Pulse model, RNA life cycle dynamics

## Abstract

The life cycle of intracellular RNA mainly involves transcriptional production, splicing maturation and degradation processes. Their dynamic changes are termed as RNA life cycle dynamics (RLCD). It is still challenging for the accurate and robust identification of RLCD under unknow the functional form of RLCD. By using the pulse model, we developed an R package named pulseTD to identify RLCD by integrating 4sU-seq and RNA-seq data, and it provides flexible functions to capture continuous changes in RCLD rates. More importantly, it also can predict the trend of RNA transcription and expression changes in future time points. The pulseTD shows better accuracy and robustness than some other methods, and it is available on the GitHub repository (https://github.com/bioWzz/pulseTD_0.2.0).

## Introduction

The response of cell stimulation is mainly manifested in the transcription, processing and degradation levels of RNA (Rabani et al., 2011; Thapar & Denmon, 2013). The dynamic combination of these processes is known as RNA life cycle dynamics (RLCD), which controls the gene expression and steady state of the cells (Zeisel et al., 2011). The cells continuously produce new RNA (pre-RNA), which will be processed into mature RNA (mRNA), and the mRNA is continuously degraded. The balance among transcription, processing and degradation rates keep cells in a steady state. However, external environmental interference, the changes in cell signal transduction and the activity transcription factor may change the rates of transcription, processing and degradation, which destroy the balance of RCLD in some cells. After a period of adjustment, cell volume growth, protein activity changes, etc., the rates of transcription, processing and degradation again reach a new equilibrium, which makes the RCLD steady in some cells again (Lopez-Maury, Marguerat & Bahler, 2008). Therefore, the identification of a continuous pattern of changes in the rates of RLCD is crucial for cell homeostasis analysis.

Currently, experimental techniques based on short pulse labeling, such as 4-thiouridine (4sU), provide the possibility to identify RLCD (De Pretis et al., 2015; Garibaldi, Carranza & Hertel, 2017; Melvin et al., 1978; Rabani et al., 2014; Sabo et al., 2014). Incorporation of thiol-containing nucleosides 4-thiouridine into nascent RNA of eukaryotic cells within a few minutes allows for non-destructive metabolic labeling of RNA. By sequencing the 4sU-labeled RNA (4sU-seq), it is possible to separate the newly generated RNA (labeled RNA) from the originally existing RNA (unlabeled pre-RNA). Rabani et al. (2011) proposed a dynamic model framework for calculating transcription and degradation, without providing software implementation. Schwalb et al. (2012) used a similar method to calculate the rates of transcription and degradation, but it lacks a calculation of the processing rates. Rabani et al. (2014) used the model of the splicing maturity process and analyzed it at the junction level. In 2015, based on the same model, a novel framework INSPEcT (De Pretis et al., 2015) was proposed, which can calculate the RLCD flexibly. In 2017, an R package of pulseR was developed based on a negative binomial distribution model (Uvarovskii & Dieterich, 2017). Although these methods identified transcriptional dynamic rates from different perspectives, they were limited to the experimental measurement time. If the measurement time is short or the number of time nodes is small, it is difficult to analyze the complete RNA life cycle. Therefore, there is an urgent need to develop a tool with the function of predicting RCLD trends, which is of great significance for RNA life cycle analysis. This function is convenient for researchers to understand the complete process of some RCLDs in cells under external stimulation, and even the complete process of cell changes which cannot be detected due to experimental limitations, which extends the current capabilities of RNA life cycle analysis tools.

Here, we developed an R package termed pulseTD that can serve as a powerful tool to identify RNA RLCD based on the pulse model. It can adequately capture the trend of RLCD, which is important to analyze the process of cells from homeostasis to new homeostasis in cell-stimulated responses. More importantly, pulseTD shows better performance in predicting RLCD and gene expression values than other methods.

## Materials and Methods

### Description of the GEO dataset

The 4sU-seq experimental datasets were obtained from GEO database (https://www.ncbi.nlm.nih.gov/geo/). The first dataset was an RNA-seq dataset from mouse DC (GSE56977). RNA was sampled from mouse DC every 15 min for the first 3 h (total 13 samples) of their response to LPS, followed by a short (10 min) metabolic marker pulse(4sU) before the sampling time point (Rabani et al., 2014). The samples of GSE59717 dataset were the infection of primary human foreskin fibroblasts with a wild-type simplex virus strain 17 with a multiplicity of infection of 10 (Rutkowski et al., 2015).

### The algorithm flow of pulseTD model

The rates of RLCD within 24 h of the pulse state were evaluated, which represented a complete RNA life cycle, and the mode of oscillation was discharged (La Manno et al., 2018). We assumed that the rates of transcription, processing and degradation conforms to the functional form of the pulse model in the pulse state. Expression value data of pre-mRNA and mRNA at any time point could be obtained by 4su-seq and RNA-seq. The parameters were estimated by using an optimization algorithm. Next, we introduced the principles of the model and the use of the software. After that we created simulation data and evaluate performance.

First, we used the R package (GenomicAlignments, GenomicFeatures, etc.) to analyze RNA-seq and 4sU-seq bam files, in order to quantify intronic (reads alignment to the intron region) and exonic (reads alignment to the exon region) in Reads Per Kilobase of exon model per Million mapped reads (RPKM), Transcripts Per Kilobase of exon model per Million mapped reads (TPM) or Fragments Per Kilobase of exon model per Million mapped fragments (FPKM) of each gene. Some methods (min–max normalization, log normalization) had been added to standardize expression profile data before evaluating RLCD. In the cell life cycle, transcription continuously generates new pre-mRNA expressed by $P\left( t \right)$. Processing converts newly generated pre-RNA into mature mRNA expressed by $M\left( t \right)$. Finally, mRNA is targeted for degradation (Moore & Proudfoot, 2009). The equation according with the process of RLCD as follows:

(1.1) $$\left\{ \matrix{ \mathop {{\rm{ }}P}\limits^ \cdot = \alpha - \gamma \hfill \cr \mathop M\limits^ \cdot = \gamma - \beta \hfill \cr} \right.$$

Among them, ${\rm {\alpha}} , {\rm{\beta}} ,{\rm {\gamma} }$ represent the expression level of synthesized, degraded and processed RNA per unit time, respectively. Since the labeling time (${t_L}$) is very short during the 4sU experimental, we assumed that RNA was not degraded during experimental labeling time. The total labeled RNA expression level ${T_L}\left( t \right)$ can be expressed as:

(1.2) $$\left\{ {\matrix{ {{{\mathop T\limits^ \cdot }_L} = {\rm {\alpha}} } \cr { {\rm{\beta}} = 0} \cr } } \right.$$

The transcription level of intracellular RNA was in a steady state without being stimulated by outside world. When some factors (transient pulse stimulation) disrupted the equilibrium, the rates of genes at different stages were changed to cushion the stimulus. After a period of time, most cells returned to a steady state due to some factors such as cell morphology and stress. As mentioned above, the change in gene expression values was mainly the result of the combination of transcription, processing and degradation. Therefore, external stimulus conditions, pulse stimuli directly affected RLCD. To this end, we hypothesized that the three processes of gene expression were transcription ${\rm {\alpha}}$, processing ${\rm {\gamma} }$, degradation ${\rm{\beta}}$ and ${\rm {\alpha}} = {\rm{\beta}}$, ${\rm {\gamma} } = {\rm {\alpha}}$ in steady state, which were broken when the external stimulus pulse was stimulated. After a period of time, it reached steady state again, and ${{\rm {\alpha}}^{\prime}} = { {\rm{\beta}}^{\prime}}$, ${{\rm {\gamma} }^{\prime}} = {{\rm {\alpha}}^{\prime}}$. The functional form of ${\rm {\alpha}} , {\rm{\beta}} ,{\rm {\gamma} }$ needed to be determined.

In this case, the rates of transcription, processing and degradation changed from ${\rm \gamma }{\sim} {\rm {\alpha}}{\sim}{{\rm{\beta}} }$ to ${\rm {\gamma }^{\prime}}{\sim}{{\rm {\alpha}} }^{\prime}{\sim}{\rm {\beta }^{\prime}}$, which could be considered as a process of RNA from a steady state to a new steady state under pulsed stimulation. So, we used the pulse model $f\left( x \right)$ (Chechik & Koller, 2009) to represent functional form of ${\rm {\alpha}} , {\rm{\beta}} ,{\rm {\gamma} }$:

(1.3) $${f_\theta }\left( x \right) = {1 \over {{h_1}}}\left( {{h_0} + \left( {{h_1} - {h_0}} \right){1 \over {1 + {e^{ - \beta \left( {x - {t_1}} \right)}}}}} \right)\left( {{h_2} + \left( {{h_1} - {h_2}} \right){1 \over {1 + {e^{\beta \left( {x - {t_2}} \right)}}}}} \right)$$

The ${\rm{\theta} }$ is the parameter vector represented by $\left( {{h_0},{h_1},{h_2},{t_1},{t_2}, {\rm{\beta}} } \right)$. ${h_0},{h_1},{h_2}$ represent the initial state rates value, the peak time rates value and the new steady state rates value. ${t_1},{t_2}$ are the maximum time for the first and second rise or fall changes, and ${\rm{\beta}}$ is the slope of the both transitions.

Taking into account the effects of existing RNA, we first estimated the global normalization factors in the model. We assumed that real mRNA $R$ levels were composed of the labeled mRNA $S$ and pre-existing mRNA $N$:

(1.4) $$R = S + N$$

Make the total mRNA observations proportional to $S$ and $N$, and the scale factors were both$w$. At the same time, we assumed that the labeled mRNA observations included two parts, labeled $S$ and unlabeled $N$, with scale factors of ${w_1}$ and ${w_2}$, respectively. Then:

(1.5) $$\left\{ \matrix{w\left( {S + N} \right) = {O_T} = T(t) \hfill \cr {w_1}S + {w_2}N = {O_L} = {T_L}(t) \hfill} \right.$$

Three scale factors are constant in one sample, and ${O_T},{O_L}$ represent observations for total RNA and labeled RNA, respectively. According to formula (1.5):

(1.6) $$\left( {{w_1} - {w_2}} \right)S + \displaystyle{{{w_2}} \over w}{O_T} = {O_L}$$

Here $P(t)$, $T(t)$ and ${T_L}(t)$ are represented as a linear combination of pulse function integrals. According to formula (1.1~1.6):

(1.7) $$\left\{ \matrix{P\left( t \right) = P\left( 0 \right) + \mathop \int \limits_0^t {f_{{{\rm{\theta} } _{\rm {\alpha}} }}}\left( t \right)dt - \mathop \int \limits_0^t {f_{{{\rm{\theta} } _\gamma }}}\left( t \right)dt \hfill \cr T\left( t \right) = T\left( 0 \right) + \mathop \int \limits_0^t {f_{{{\rm{\theta} } _{\rm {\alpha}}}}}\left( t \right)dt - \mathop \int \limits_0^t {f_{{{\rm{\theta} } _ {\rm{\beta}} }}}\left( t \right)dt \hfill \cr {T_L}\left( t \right) = {\displaystyle{{{w_2}} \over w}T\left( t \right)} + \left( {{w_1} - {w_2}} \right)\mathop \int \limits_{t - {t_L}}^t {f_{{{\rm{\theta} } _{\rm {\alpha}} }}}\left( t \right)dt \hfill} \right.$$

Where $P\left( t \right),T\left( t \right),{T_L}\left( t \right)$ are the observation data of the measurement time node, ${t_L}$ is the labeled time, ${{\rm{\theta} } _x} = \left( {h_0^x,h_1^x,h_2^x,t_1^x,t_2^x,{ {\rm{\beta}} ^x}} \right)$, so the parameter vector $\; X = \left( {{{\rm{\theta} } _{\rm {\alpha}} },{{\rm{\theta} } _{\rm {\gamma} } },{{\rm{\theta} } _ {\rm{\beta}} }} \right)$. The total objective function is

(1.8) $$J\left( X \right) = \displaystyle{1 \over 2}\mathop \sum \limits_{j = 0}^{{t_{end}}} {\left( {{{\hat P}_j} - N{P_j}} \right)^2} + {\left( {{{\hat T}_j} - N{T_j}} \right)^2} + {\left( {{{\hat T}_L}_j - N{T_L}_j} \right)^2}{\rm \; }$$

Among them $\hat P,\hat T,{\hat T_L}$ are the model prediction value and $NP,NT,N{T_L}$ are the standardized observation data. Therefore, ${\rm {\alpha}} , {\rm{\beta}} ,{\rm {\gamma} }$ can be solved by the following constrained optimization problem:

$$X = \arg minJ\left( X \right)$$

$${\rm s}.{\rm t}.\,\,X\,\gt\,0,$$

$${\rm h}_0^{{\rm {\alpha}} }{\sim}{\rm h}_0^ {\rm{\beta}} {\sim}{\rm h}_0^{\rm {\gamma} } ,$$

$${\rm h}_2^{{\rm {\alpha}} }{\sim}{\rm h}_2^ {\rm{\beta}} {\sim}{\rm h}_2^{\rm {\gamma} } ,$$

First, we evaluated the global normalization factor using a gradient descent method with random initialization parameters. We minimized the objective function by using the nlminb method, which was available in the stats R packages. The best fit result was chosen from the results of 100 (default) random initial values. At the same time, the chi-square test of goodness of fit was used to verify the statistical validity of pulseTD.

### The interpretability of the model output

Here, the rates of transcription, processing and degradation were defined as the expression level of RNA transcribed, processed or degraded per unit time (unit: RNA/min or RNA/hour). In order to increase the interpretability of pulseTD and make it easier to compare with other tools, a conversion method was applied to pulseTD output. Let ${\rm {\gamma} } ={{\rm {\gamma} } ^k}P$ and ${\rm{\beta}} = { {\rm{\beta}} ^k}\left( {T - P} \right)$ at any time *t*. We assumed that the processing rates were directly proportional to the pre-mRNA expression and the ratio was ${{\rm {\gamma} } ^k}$. Similarly, degradation rates were directly proportional to mature mRNA expression value, the ratio was ${ {\rm{\beta}} ^k}$. According to formula (1.1):

(1.9) $$\left\{ {\matrix{ {\mathop P\limits^ \cdot = {\rm {\alpha}} - {{\rm {\gamma} } ^k}P} \cr {\mathop T\limits^ \cdot = {\rm {\alpha}} - { {\rm{\beta}} ^k}\left( {T - P} \right)} \cr } } \right.$$

This was similar to previous researches (De Pretis et al., 2015; Rabani et al., 2011, 2014; Sun et al., 2012). ${\rm {\alpha}}$ represents the transcription rates in units of mRNA/min. Where $\mathop P\limits^ \cdot$ and $\mathop T\limits^ \cdot$ represent the derivatives of the functions *P*(*t*) and *T*(*t*) at time *t*, respectively. And they are functions of time *t*. Here we used ${\rm {\alpha}} ,{{\rm {\gamma} } ^k},{ {\rm{\beta}} ^k}$ to compare with other methods, and it was easy to explain.

### Generation of simulation data

To evaluate the effectiveness of the model, we generated simulation data for 1,000 genes by randomly drawing from a specific distribution. Transcription, processing, and degradation rates were first generated, as well as randomly generated scale factors. The simulation expression value was then created based on the rates using Runge–Kutta method.

First of all, based on previous researches, we knew that the half-life of RNA was considered to follow a normal distribution (Friedel et al., 2009). Next, we determined the distribution of the transcription rates based on the mean ($\rm \mu$) and variance ($\rm \sigma$) of the observations. And the transcription rates were randomly extracted t from the normal distribution $N\left( {\rm \mu ,{\rm \sigma ^2}} \right)$. We had expected to determine the degradation rates in the same way. However, the degradation rates and the transcription rate were dependent. To simulate this dependency, we evaluated the correlation between transcription and degradation rates at any time based on the pearson correlation coefficient, which was ${k_t}$. Therefore, the degradation rates approximately obeyed the $N\left( {{\rm{\mu }}/{{\rm{k}}_{\rm{t}}},{{\left( {{\rm{\sigma }}/{{\rm{k}}_{\rm{t}}}} \right)}^2}} \right)$ distribution. Similarly, processing rates were randomly drawn in the same way. Finally, we also randomly generated global scale factors, which were used to simulate existing and newly generated RNA.

After determining all rates, we used the fourth-order Runge–Kutta method of the R package (deSolve) to evaluate the expression levels of pre-mRNA, mature mRNA and labeled RNA as a simulation data set. Among them, the initial value of the expression value was randomly selected from the distribution of the observed data.

In general, we determined the rate distribution based on the experimental data and generated simulated data parameters based on the mean and variance of the rate. Here, the time range of the experimental analysis was 0–180 min, sampling was performed every 15 min, and the 4sU marking was performed 10 min before sampling. Finally, we got a simulation data set of 1,000 genes.

## Results

### Software framework and description

**Figure 1:**
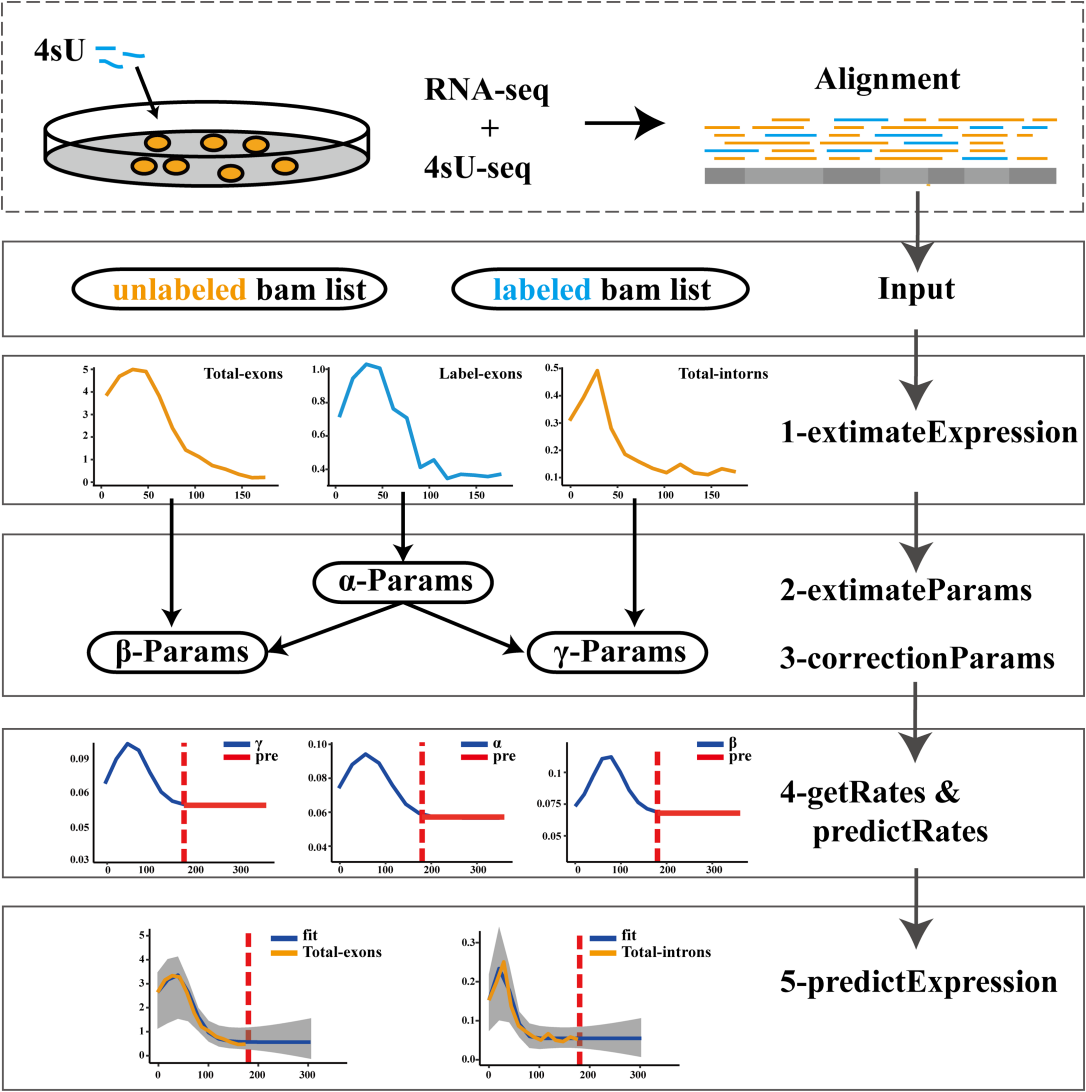
Flowwork corresponding software function.

The pulseTD combines the pulse model to predict the steady state of the RLCD. We defined the rates of transcription processing and degradation as a pulse function which had 6 parameters, a total of 18 parameters. To standardize RNA expression levels, additional global scale factors needed to be evaluated. For the evaluation of each gene, 100 random initializations were required. We used a multi-threaded approach to reduce runtime. The software supports different ways to evaluate expression levels such as counts, RPKM, TPM, FPKM. The workflow of pulseTD software is as follows (Fig. 1): (i) The RNA-seq and 4sU-seq data are aligned to the reference genome, and the results are used as the input files of the software. Expression values of pre-RNA, mRNA and labeled-RNA can be calculated in the form of RPKM, corresponding to the R function named extimateExpression. (ii) Subsequently, the expression profile is used to optimize the parameters of the pulse model. This is an optimization problem with six parameters, which are determined by MSE (minimizing the mean square error). The extimateParams function can estimate the pulse parameters of the transcription, processing and degradation rates. (iii) The parameters can be re-estimated using the correctionParams function because of the influence of random initial values. (iv) Next, transcriptional dynamic rates are solved flexibly, including the rates of transcription, processing and degradation, or the steady state rates are predicted. Complete guide reference documentation.

### Compared with other methods

Several published studies had revealed RNA RLCD from different perspectives. We compared the following common software tools: INSPEcT (De Pretis et al., 2015), DRiLL (Rabani et al., 2014), DTA (Schwalb et al., 2012) and pulseR (Uvarovskii & Dieterich, 2017). pulseTD, INSPEcT and DRiLL provide evaluation functions for gene expression and processing rates, except for DTA and pulseR. The encoding of DRiLL is MATLAB, which requires a linux system and has a large operational limit. In terms of model, pulseTD directly applies the pulse model to different stages of RLCD, making the calculation of RLCD continuous. Although the pulse model was also used in INSPEcT and DRiLL, the purpose was to fit the expression pattern. Most importantly, pulseTD has the ability to predict RLCD (Table 1). Overall, pulseTD improves accuracy between biological repetitions and enhances the performance of evaluating low expression data. At the same time, it adds the ability to predict steady state. The assumption that the transcription rate is constant within the 4sU labeling time is abandoned in the solution process, and dynamic rates and gene expression values can be predicted at some future time points.

**Table 1:** Detailed description of the software function.

	pulseTD	INSPEcT	DRiLL	DTA	pulseR
Code language	R	R	MATLAB	R	R
Gene expression	1	1	1	0	0
Processing rates	1	1	1	0	?
Prediction	1	0	0	0	0
Continuity	1	0	0	0	0

**Note:**

1, means available; 0, means unavailable; ?, representative unknown.

### Performance analysis

**Figure 2:**
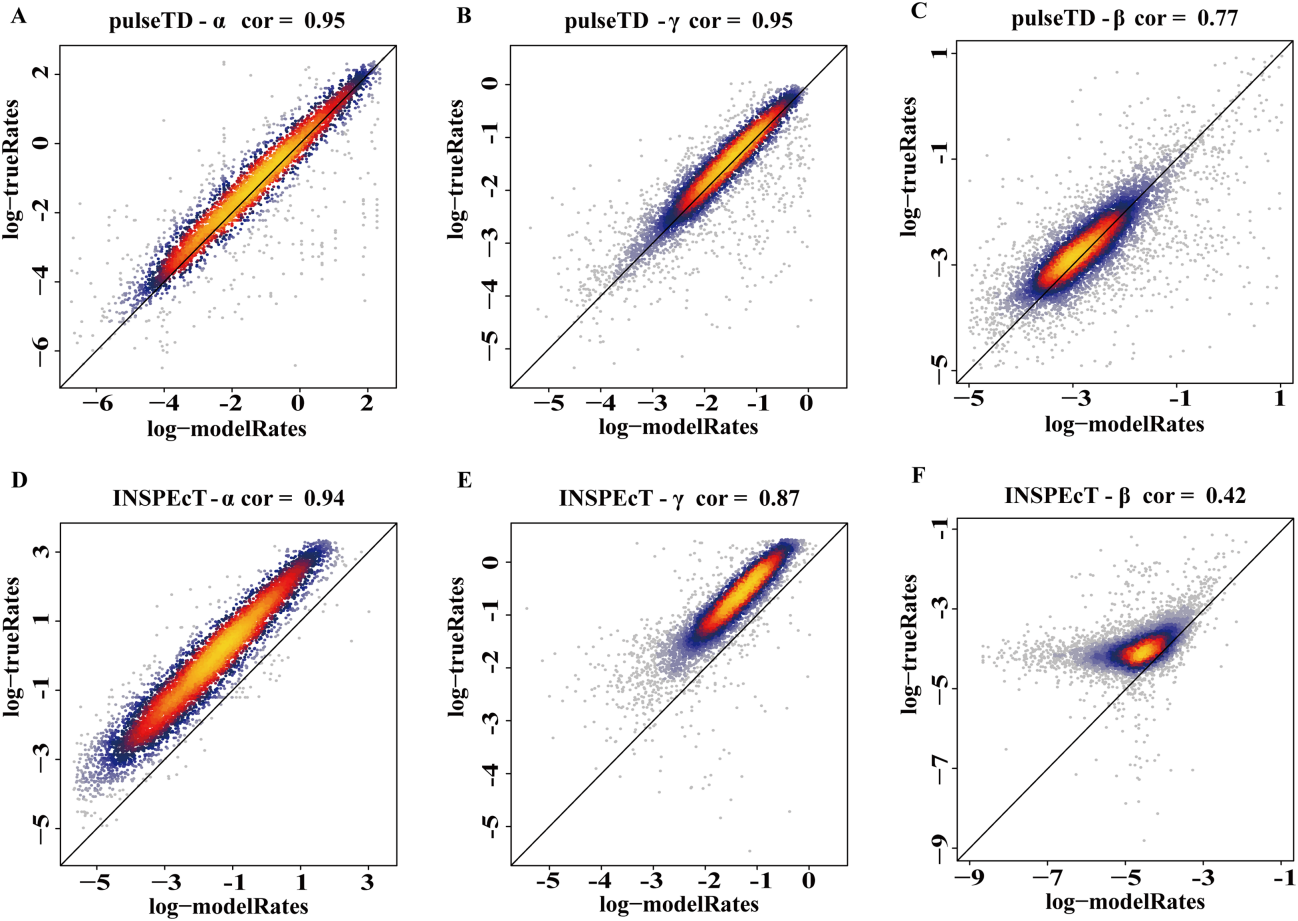
pulseTD and INSPEcT accuracy analysis and comparison. (A–C) Scatter plots of the correlation between the RLCD rates and the true rates evaluated by pulseTD, which represent the transcription, processing and degradation rates, respectively. The *x*-axis and *y*-axis are the logarithm of the simulated and real rates values. The color is closer to yellow, and the density of scatter is larger. (D–F) Scatter plots of the correlation between the RLCD rates and the true rates evaluated by INSPEcT, which represent the transcription, processing, and degradation rates, respectively. The *x*-axis and *y*-axis are the logarithm of the simulated and real rates values. The color is closer to yellow, and the density of scatter is larger.

**Figure 3:**
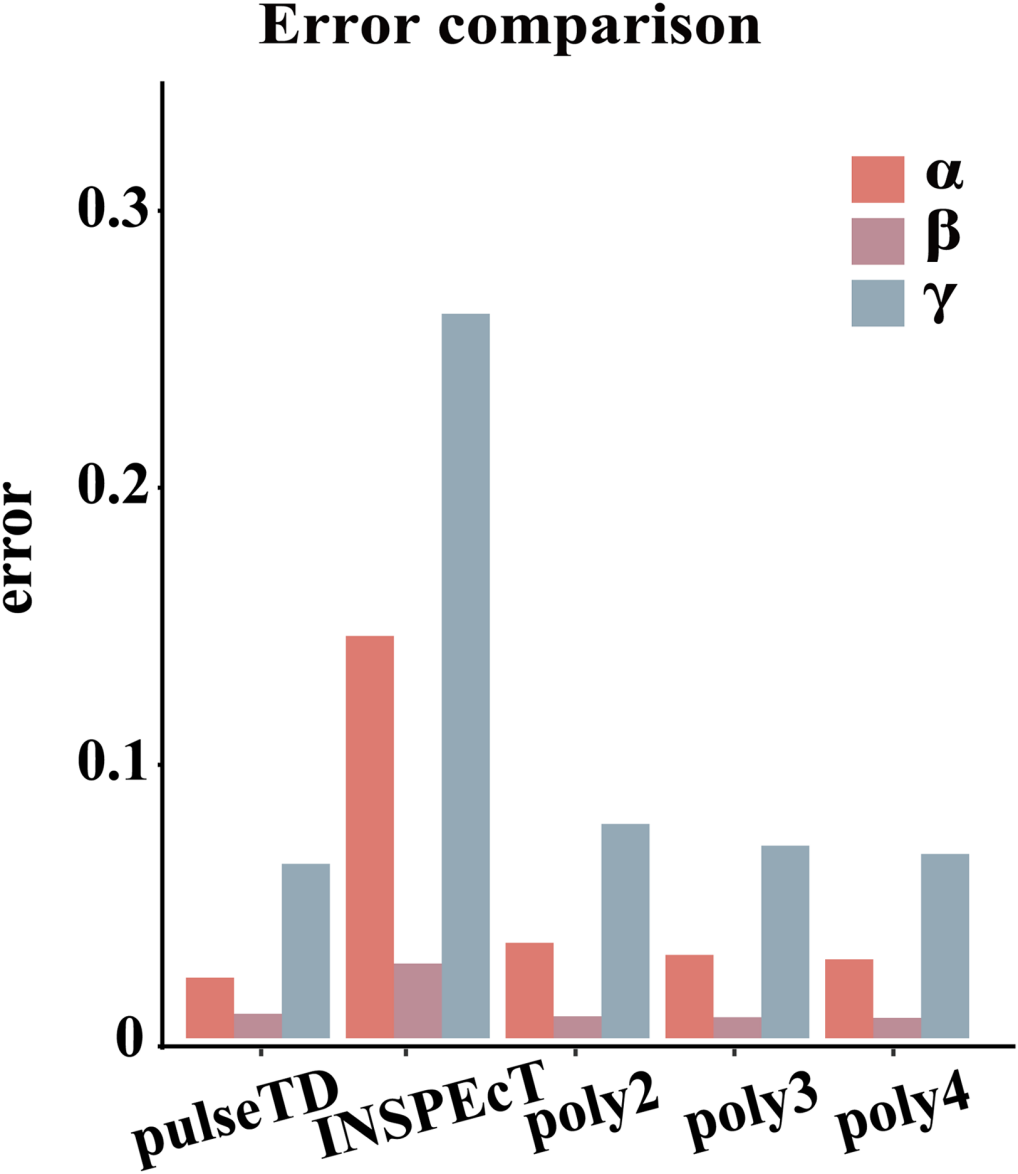
Compare RCLD errors between different software. The bar chart with error comparison of different methods, including pulseTD, INSPEcT, second-order (poly2), third-order (poly3) and fourth-order (poly4) polynomials. The *y*-axis is the error value of the RCLD, and α is the transcription rates, β is the degradation rates, and γ is the processing rates.

The efficacy of pulseTD was evaluated on the simulation dataset. We calculated the Pearson correlation coefficient (PCC) for pulseTD between real and simulated transcriptional dynamic rates, and found that the PCC values for transcription, processing and degradation rates were 0.95, 0.95 and 0.77, respectively (Figs. 2A–2C). The PCCs for the transcription, processing, and degradation rates of INSPEcT were 0.94, 0.87 and 0.42, respectively. However, the rates of RCLD evaluated by INSPEcT were less than the true rates (Figs. 2D–2F). The MSE between real and simulated RCLD rates were calculated using pulseTD, INSPEcT and curve-fitting methods. The results showed that the MSE value of pulseTD was <0.1 (Fig. 3). These results suggested that pulseTD was accurate in assessing the RCLD rates.

**Figure 4:**
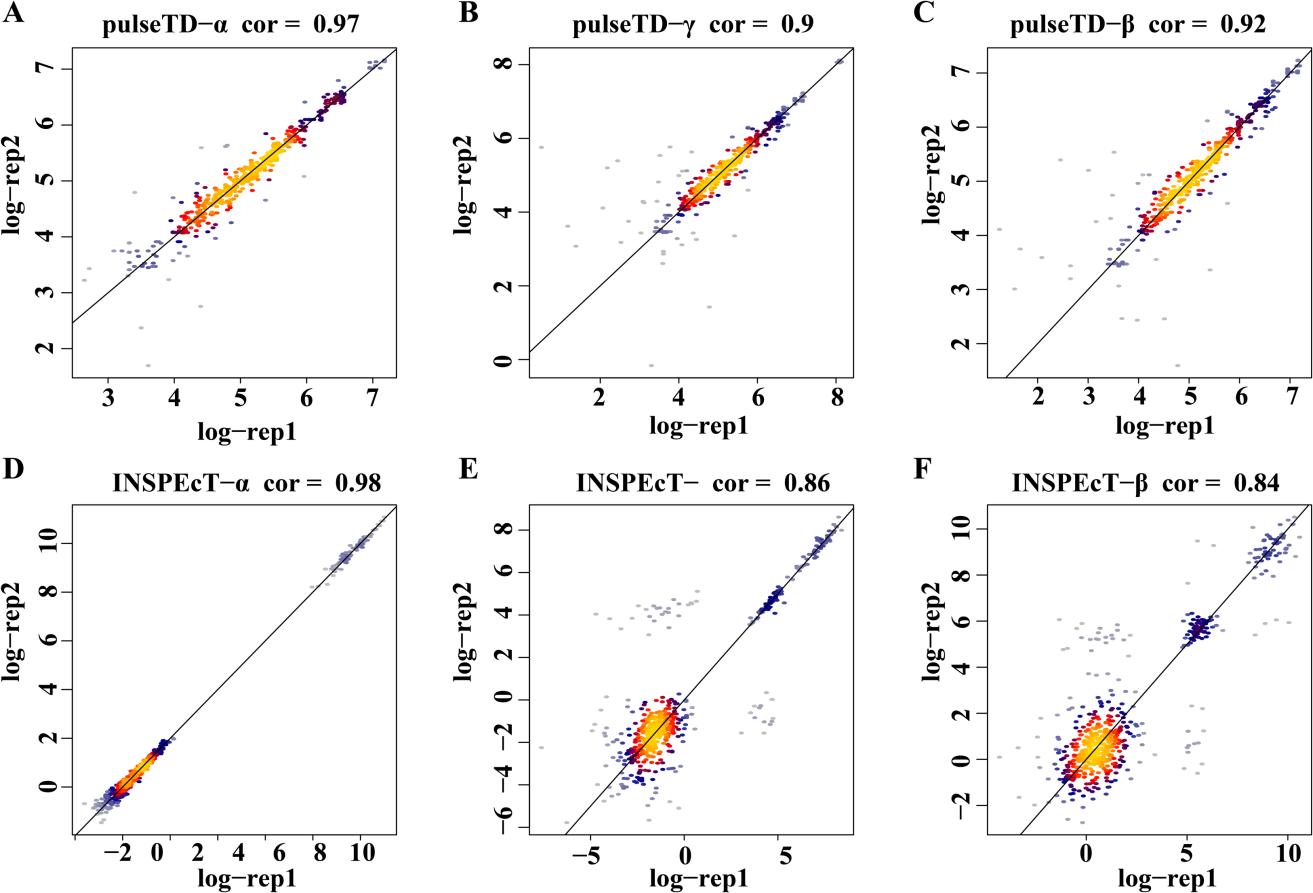
Accuracy analysis and comparison based on the simulation data generated by INSPEcT. (A–F) Scatter plots of the correlation. The *x*-axis is the logarithm of replication-1, the *y*-axis is the logarithm of replication-2. The color is closer to yellow, and the density of scatter is larger. (A–C) is using pulseTD software and (D–F) is using INSPEcT software.

To eliminate the bias of simulation data, simulation data produced by INSPEcT was used to evaluate the performance of pulseTD. We used the INSPEcT tool to generate two biological replications dataset, which contained 10 time nodes. The correlation of total RNA expression dataset was 0.98. Then, we used pulseTD to evaluate the RCLD of the biological replications, where the correlations of transcription, processing and degradation rates were 0.97, 0.90 and 0.92 (Figs. 4A–4C). As a comparison, we used INSPEcT to evaluate the RCLD of the simulation data. The correlations of transcription, processing, and degradation rates were 0.98, 0.86 and 0.84 (Figs. 4D–4F). In general, pulseTD had shown good performance in different simulation data.

**Figure 5:**
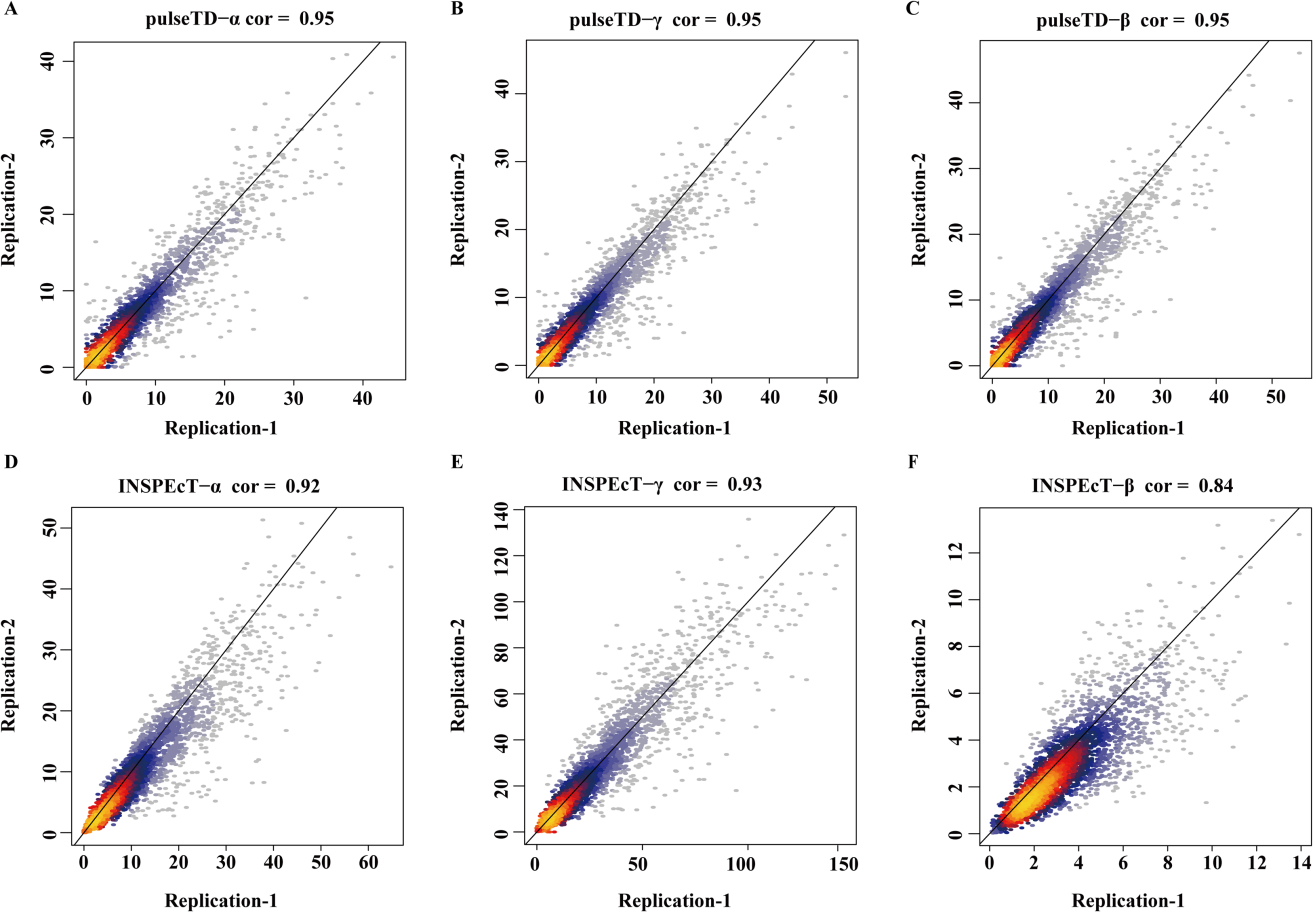
The correlation of biological duplicate data. (A–F) Scatter plots of the correlation. The *x*-axis is the replication 1, the *y*-axis is the replication 2. (A–C) is using pulseTD software and (D–F) is using INSPEcT software.

**Figure 6:**
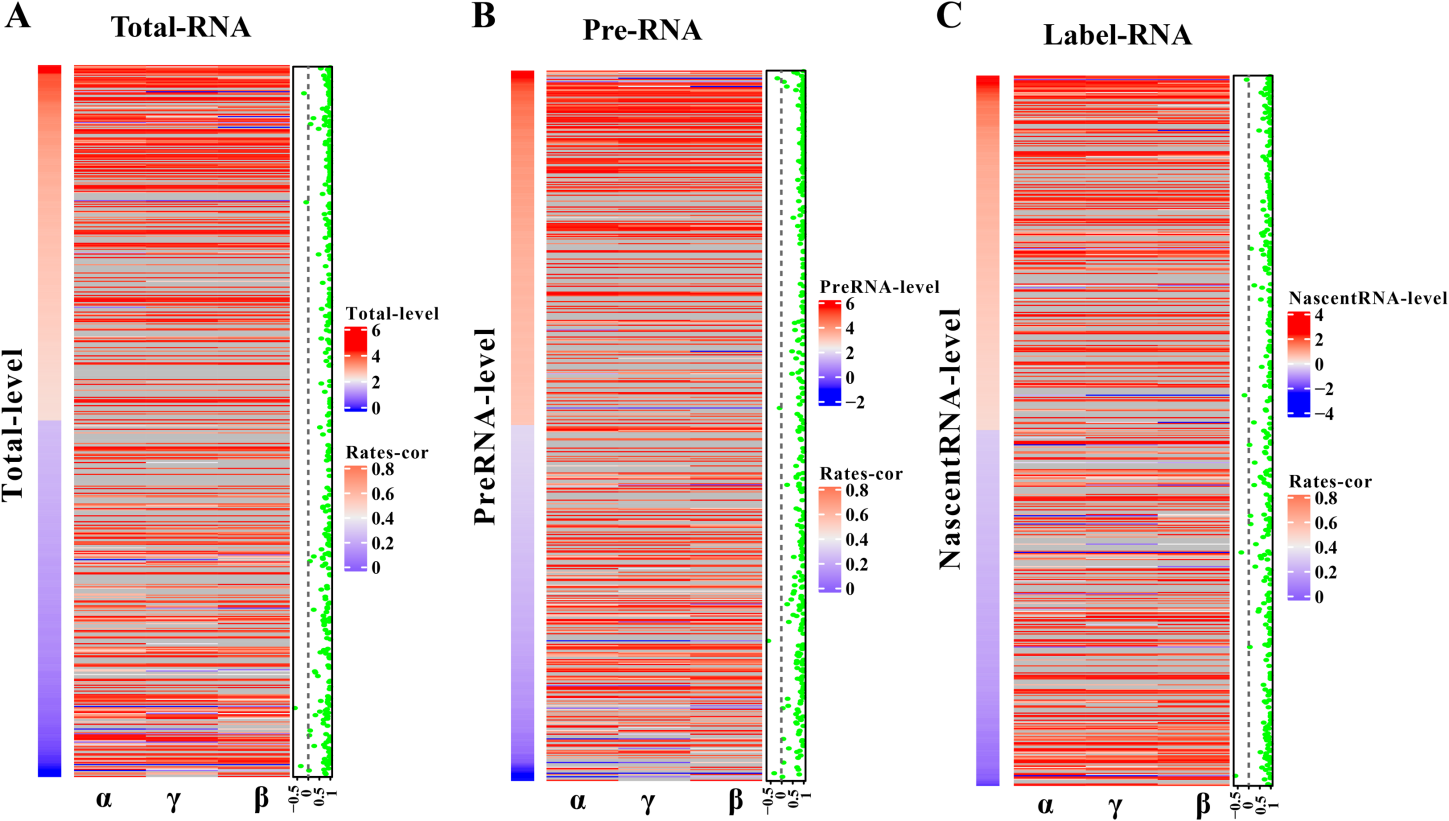
Influence of gene expression level on model optimization. (A–C) Heat maps of total RNA, pre-RNA and labeled RNA, respectively. (A) A heat map of expression levels. (B) The respective correlation of transcription, processing and degradation rates between two biological replicates. (C) A scatter plot of the mean correlation of transcription, processing, and degradation rates.

The robustness of pulseTD was tested using two biologically repeated gene expression datasets from GEO (GSE59717). The PCC values of transcription, processing, and degradation rates between replications were 0.95, and the RCLD rates distribution also showed high consistency (Figs. 5A–5C). This reflected high robustness of pulseTD. For comparison, the PCC values of INSPEcT were calculated using the same data, they were 0.92, 0.93 and 0.84 (Figs. 5D–5F). The PCC for its degradation rates was low, which might be due to more outliers, during the evaluation process. And its density distribution produced a large deviation. pulseTD showed higher stability in biological replicate data. At the same time, in order to judge the impact of expression levels of gene on software performance, we calculated the mean expression levels of the total exons, total introns and labeled exons of each gene during the detection time and ranked the mean. The first 1/3 genes were considered to be low expression, and the last 1/3 genes were considered to be high expression. Both of them were used to evaluate RCLD. The PCC value of degradation rates and processing rates obtained by INSPECT were 0.58 and 0.68, but the PCC value of pulseTD remained stable. This showed that pulseTD had a higher tolerance for poor quality data and the evaluation results were more stable. We also explored the effects of pre-RNA, total RNA and labeled RNA expression levels on model optimization. The correlation of the transcription, processing and degradation rates of each gene was calculated between the two biological replicate data sets. At the same time, we sorted them according to the mean expression levels of the total exons, total introns and labeled exons, respectively. We found that the number of genes with an average correlation greater than 0.5 was 96.21% and overall showed a high correlation (Figs. 6A–6C). This indicated the expression levels of total RNA, pre-RNA and labeled RNA had little effect on the optimization of the model.

### Predict RLCD at unknown time nodes

**Figure 7:**
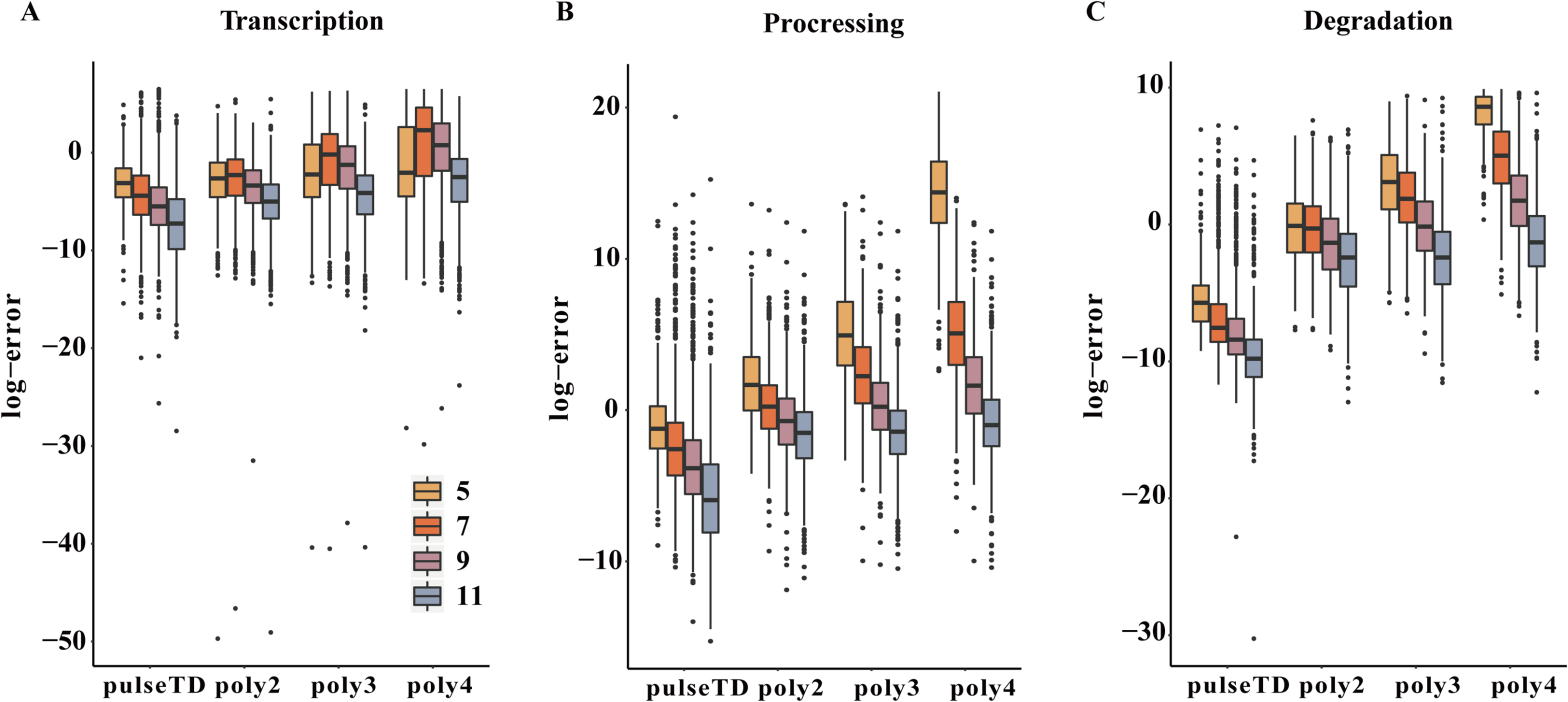
Comparison of the efficacy of different models for predicting the RCLD. (A–C) Box plots for the comparison of prediction errors of different methods, including pulseTD, INSPEcT, second-order (poly2), third-order (poly3) and fourth-order (poly4) polynomials. The smaller the error value, the higher the accuracy of the prediction. The *y*-axis is the negative logarithm of the mean square error between the predicted and true values, and 5, 7, 9 and 11 in the legend represent the number of experimental measurement time points, respectively.

The pulseTD can predict dynamic transcription rates and gene expression because of its pulsed model characteristics. To evaluate prediction effectiveness, we divided the GEO (GSE56977) dataset into two parts. We selected the first 5, 7, 9 and 11 time points from 13 measurement time points as training samples (Assuming they are experimental measurement data) to estimate the rates of RCLD and used the remaining time points (test samples) for prediction. We estimated and predicted the rates of RCLD based on the training samples by using pulseTD and some other methods. Then, the MSE values between the predicted results and the test samples were calculated to evaluate the prediction performance. The results showed that pulseTD had the lower average MES values of RLCD rates than other methods (Figs. 7A–7C). These results showed that pulseTD had good prediction capabilities. We also found that as the training time points increase, the MSE values gradually decreased. When estimating RLCD, we recommend increasing the number of measurement time points in the experiment in order to have more accurate predictions.

## Conclusions

In the field of bioinformatics, it is important to accurately identify the rates of RLCD and predict transcriptional stability. Few programs can identify the rates of RLCD, and none can provide predictions of the dynamic rates and steady state of RLCD. Here we use 4sU-seq and RNA-seq technology to analyze and predict RLCD. In summary, based on the pulse model, combined with the biological significance of RNA life cycle, we developed the R package named pulseTD. It only needs the alignment files of 4sU-seq and RNA-seq to calculate the expression value and the pulse model parameters in a simple manner. Here, we recommend using min–max normalization when comparing experiments with different conditions to remove the dimension and logarithmic normalization when analyzing single experimental data to narrow the range of values. It can easily evaluate the RLCD at any time points. More importantly, it can predict the trend and the steady state of transcriptional dynamic rates. It has better accuracy and robustness than other methods. You can get source code on GitHub (https://github.com/bioWzz/pulseTD_0.2.0).

## Additional Information and Declarations

### Data Availability

The following information was supplied regarding data availability: The code is available on GitHub: https://github.com/bioWzz/pulseTD_0.2.0.
